# A review of the clinical value of mechanical ventilators and extracorporeal membrane oxygenation (ECMO) equipment

**DOI:** 10.1016/j.ipemt.2024.100031

**Published:** 2024-12

**Authors:** David Stell, Dr Man Ting Kwong, Robert Megwa, Dr Tom Bashford, Dr. Emmanuel Akinluyi, Prof. P. John Clarkson

**Affiliations:** a Engineering Design Centre, Department of Engineering, University of Cambridge, Cambridge, CB2 1PZ, United Kingdom; b Department of Medical Physics and Clinical Engineering, Guy's and St Thomas’ NHS Foundation Trust, London, SE1 9RT, United Kingdom; c Adult Critical Care, Guy's and St Thomas’ NHS Foundation Trust, London, SE1 9RT, United Kingdom; d International Health Systems Group, Engineering Design Centre, Department of Engineering, University of Cambridge, Cambridge, CB2 1PZ, United Kingdom; e Department of Anaesthetics, Cambridge University Hospitals NHS Foundation Trust, Cambridge, CB2 0QQ, United Kingdom; f Cambridge Public Health Interdisciplinary Research Centre, Cambridge, CB2 1PZ, United Kingdom; g Research Department of Imaging Physics & Engineering, School of Biomedical Engineering and Imaging Sciences, King's College London, London, WC2R 2LS, United Kingdom

**Keywords:** Mechanical ventilation, Extracorporeal membrane oxygenation, Clinical Engineering, medical equipment management, prioritisation, value

## Abstract

Acute healthcare providers operate large, diverse medical equipment inventories. Resources for managing these inventories is frequently scarce so must be prioritised such that maximum benefit is conferred per unit of expenditure.

This review identifies publications which have discussed the clinical value conferred by mechanical ventilation (MV) and by extra corporeal membrane oxygenation (ECMO). Respectively, mechanical ventilators and ECMO units are necessary to deliver these therapies. Systematic searches for publications which discuss the clinical value conferred by MV and by ECMO were conducted.

The identified articles included reviews, prospective studies, retrospective studies, and models. Most presented findings in terms of the cost-effectiveness ratio. The patient populations studied, and analytical methods used varied widely. The clinical value conferred by MV varied with dependencies on several factors including the age- and disease- profile of the patient population. It was not possible to infer these dependencies from the literature which exists for ECMO.

More relevant literature existed for MV, the more mature technology, than did for ECMO. The ECMO literature also tended to be more recent and included more modelling studies and fewer prospective studies. The data extracted could inform estimates of the clinical value likely to be delivered by mechanical ventilators operated by a specific institution. Estimates for ECMO are likely to carry greater uncertainty than those for MV.

## Highlights

• MV & ECMO clinical value estimates exist in health economics articles, among others.
• Conferred clinical value varies depending on patient population characteristics.
• Higher quality data and more sub-group analyses exist for MV than for ECMO.
• Institution-specific clinical value estimates will be less certain for ECMO than MV.

## 1 Introduction

### 1.1 Medical Equipment Value

Modern acute hospitals operate large, diverse medical equipment inventories. These require ongoing maintenance, periodic replacement of aging units, and intermittent adoption of new technologies. These activities require funding and manpower, and available resources are seldom enough to service all requests. Prioritisation decisions, where resources are allocated for some requests and not others, therefore form a routine part of medical equipment management.

The aim of these decisions should be to maximise the clinical value delivered with the resources available. This requires estimating the clinical value that a unit of medical equipment will deliver. These estimates are challenging. When used in an acute hospital setting, medical equipment exists in complex systems composed of interdependent elements. Predicting the impact of adding or removing any element presents a significant analytical challenge. The challenge is less when the equipment is required for the delivery of care. In these cases, the clinical value lost by removing the equipment corresponds to the entire clinical value conferred by the care.

As examples, mechanical ventilators and extra corporeal membrane oxygenation (ECMO) systems are necessary for the delivery of mechanical ventilation (MV) and ECMO therapies. Both therapies can treat critically ill patients with compromised respiratory function. ECMO can also replace cardiac function. This review seeks to collate and summarise published clinical value estimates for these equipment types, such that they could be used to inform equipment management decisions by individual healthcare providers.

Publications which consider the clinical value of MV and ECMO are reviewed. Quantitative figures for the clinical value conferred per patient treatment are extracted, together with those for clinical value per unit duration of treatment (given that treatment duration may vary between patients). The review also considers other dependencies which affect the clinical value conferred by MV and ECMO.

### 1.2 MV and ECMO technology

MV is a mature technology, first deployed in the early 1950s to combat the polio epidemics of that period [1]. The world's first intensive care units, opened in 1953, were created for MV patients [2]. Mechanical ventilators have been a mainstay of intensive care unit (ICU) care since then but have continued to see innovation, with new ventilation modes and other novel techniques to improve outcomes.

ECMO also has a long history. The first extracorporeal blood oxygenation devices were used to increase the time patients could survive cardiac arrest during cardiac surgery. Oxygenation was achieved by direct contact between blood and gaseous oxygen. This caused blood cell damage, limiting the maximum duration of use. ECMO was a development of this technology; placing a semi-permeable membrane between the blood and oxygen reduced blood cell damage allowing long-term use of the technique. [3]

ECMO's first successful use in humans was for a respiratory failure patient in 1972 [4]. However, for many years researchers failed to demonstrate superiority to conventional MV in randomised controlled trials (RCTs) [5,6], so clinical adoption was slow. The first RCT to return positive results was for neonatal patients in 1997 [7,8]. A subsequent 2009 RCT returned positive results for adult patients [9,10], stimulating rapid global adoption of ECMO technology [11].

The most widely used ECMO variant is venovenous ECMO (VV-ECMO), in which blood is extracted from a vein, passed through an extracorporeal oxygenator, and returned to another vein [12]. A related technique is extracorporeal carbon dioxide removal (ECCO_2_R) whose purpose is to remove carbon dioxide from the blood while oxygenation occurs predominantly in the lungs. Use of ECCO_2_R permits lower ventilator tidal volumes than would otherwise be possible and requires much lower extracorporeal blood flow rates than true VV-ECMO (and consequently lighter clinical oversight) [13]. Both VV-ECMO and ECCO_2_R augmented MV can serve as alternatives to conventional MV.

In venoarterial ECMO (VA-ECMO) inflow to the extracorporeal system is from a vein but outflow is to an artery. This allows the ECMO pump to replace the function of the heart. VA-ECMO can treat cardiogenic shock, where a patient's cardiac output is not sufficient to sustain life [12]. When used as an emergency treatment for cardiac arrest, VA-ECMO is known as extracorporeal cardiopulmonary resuscitation (ECPR) and can be an alternative to conventional cardiopulmonary resuscitation (CPR).

Although now widely used, ECMO's clinical effectiveness is subject to ongoing research with at least twelve RCTs being published in this area since 2018 [[14], [15], [16], [17], [18], [19], [20], [21], [22], [23], [24], [25]]. This literature is relevant to the present discussion, as any assessment of a technology's clinical value presupposes that the technology is clinically effective.

### 1.3 Quantification of clinical value

Within health economics, clinical value is conventionally expressed in units of quality assessed life-years (QALYs). A single QALY is calculated by multiplying a duration of life (in years) by a utility multiplier representing the quality of life during that time. The utility multiplier may vary between 0 (equivalent to death) and 1 (equivalent to perfect health). Quality assessed life expectancy (QALE) is the total number of QALYs a person is forecast to experience in their remaining life. Life expectancy, which does not account for quality of life, has sometimes been used in place of QALE, particularly in older research.

QALE is most often calculated as part of cost-effectiveness analyses (CEA). A key output of these studies is the cost per QALY quantity, known as the cost-effectiveness ratio (CER). They may also publish the difference between the CERs for different technologies, a quantity known as the incremental cost-effectiveness ratio (ICER). The present review is not specifically concerned with CER figures, being interested solely in the denominator of the CER equation. However, given that QALE calculations form a necessary part of CER calculations, CEA studies are of particular interest to the review.

## 2 Methods

### 2.1 Literature search

The PubMed, Medline, EMBASE and Web of Science indexes were queried for all historic articles including terms related to MV or ECMO, and terms related to clinical value. Further searches were conducted on the Cost Effectiveness Analysis registry maintained by the Tufts Medical Centre [26] for articles including terms related to MV or ECMO. The search terms used are given in the supplemental material.

Articles whose analysis was limited to quality of life or which considered patients’ inpatient stays only were excluded. Review articles were considered for inclusion as well as original research articles.

A single reviewer (DS) screened each unique abstract and excluded those not relevant. Records which were not excluded were sought for retrieval. Each retrieved article was reviewed by the principal researcher and those eligible were included in the review.

### 2.2 Evaluation of quality

A checklist (available in the supplemental material) was used to assess the quality of each original research article. The evaluation only considered aspects relevant to the determination of clinical value, other aspects were neglected (e.g., those relevant solely to the evaluation of costs, or to clinical effectiveness).

The factors considered included sample size, use of a comparator group, whether analysis was based on data from a single patient cohort or drawn from studies of different cohorts, whether QALE values were presented, whether inclusion criteria could be evaluated before treatment commenced (as opposed to factors known only at the termination of treatment, e.g., treatment duration), the proportion of patients whose length-of-life post-treatment was directly observed (i.e., who died during the follow-up period), the technique used to estimate quality of life and whether it conformed to the prevailing gold standard [27], whether relevant study population characteristics (e.g., age and disease profile) were described, whether therapy duration data was presented, the horizon used for QALE assessment, whether life expectancy assessment accounted for patient population characteristics (e.g., whether it accounted for the illness's impact on life expectancy, rather than assuming survivors had life expectancy equivalent to the general population), whether a discount rate was applied when assessing the value of future life-years and, for models, whether a sensitivity analysis was presented.

### 2.3 Extraction of qualitative data

Where articles identified relevant dependencies (i.e., factors predictive of the clinical value conferred by MV and ECMO), these were considered within the analysis. Characteristics of the patient population likely to be predictive of clinical outcome (such as age and diagnostic profile) were extracted where available.

### 2.4 Extraction of quantitative data

For each original research article, values for QALE and/or life expectancy were extracted where available. Where unavailable, these values were derived from other data if possible.

Where articles quantified clinical value (in terms of QALE or life expectancy) together with therapy duration, the ratio of mean clinical value to mean duration was calculated. Where therapy duration was not presented, length-of-stay was used if available. Length-of-stay will usually overestimate the duration of therapy, so its use will tend to underestimate the clinical value conferred. Where length-of-stay has been used rather than duration, this is noted in the results.

Frequency distributions for length-of-stay and therapy duration are usually positively skewed. Use of the median and quartiles, rather than the mean and standard deviation, to describe the distribution is therefore appropriate. However, calculation of clinical value per unit time requires use of mean (not median) values. Where the mean was unavailable, the skew normal, gamma and Weibull distributions defined by the median and quartile figures were calculated (algorithm available in the supplemental material). The distribution which best matched the data was then used to estimate the mean. Where the median was given in isolation (without the quartiles), it was used as an order-of-magnitude estimate of the mean. For positively skewed distributions the median is less than the mean, so use of the median will overestimate the clinical value conferred per unit time. Where figures other than the true mean have been used, this is noted in the results.

## 3 Results

### 3.1 Literature search

**Fig. 1:**
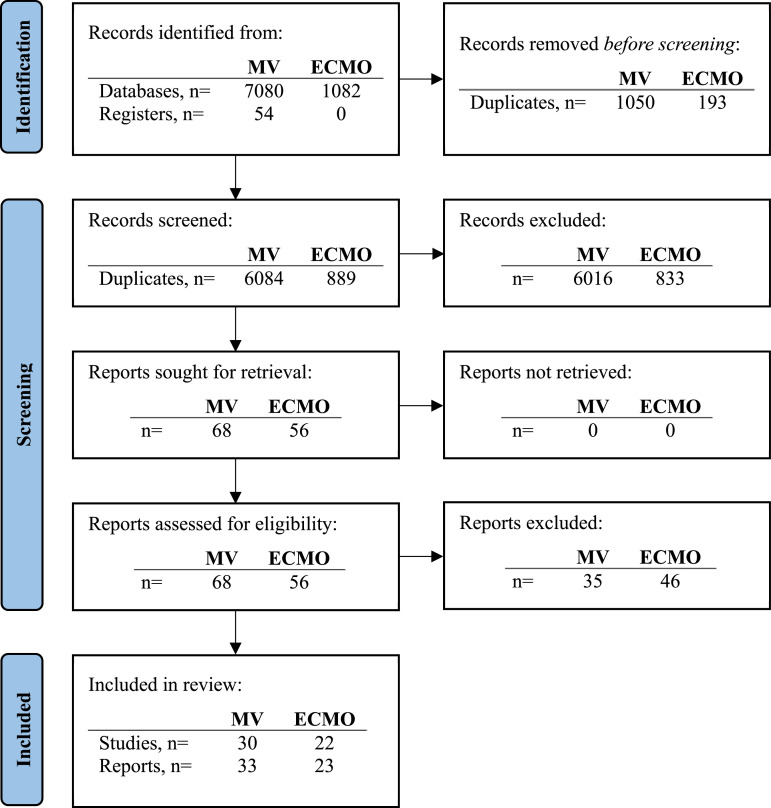
Results of the literature search for MV and ECMO publications.

The results of the literature search are summarised in Fig. 1. The included review articles are described in Table 1 and the included original research articles are outlined in Table 2.

**Table 1:** Included review articles.

Reference	Type	Technology	Summary
Rosen & Bone (1988) [32]	Non-systematic review	MV	Considered several previous studies into MV in an acute setting, concluding that care involving MV is expensive, and outcomes are poor. The review went on to consider the provision of ongoing MV for patients who require long term ventilatory support. Most of the discussion in this article consisted of a discussion of provision of prolonged MV in settings other than acute hospitals.
Shorr (2002) [33]	Non-systematic review	MV	Noted that intensive care is expensive because of both high fixed costs and high marginal costs (e.g., use of high-cost pharmaceuticals and imaging). The author also noted that demand for this care is increasing. The review considered the uncertainty inherent in any CEA, particularly that associated with assessing the costs of care. Three ICU CEA studies were critiqued and summarised. The author also considered the effect that intensive care unit organisation can have on cost-effectiveness noting that some researchers had concluded that certain intensive care unit structures simultaneously reduce costs and improve outcomes.
Carson (2006) [34]	Non-systematic review	MV	Focussed on the outcome of patients who receive prolonged MV. The author noted that defining “prolonged” is problematic, and favoured definitions which capture only patients whose MV durations are statistical outliers, and which avoid capturing patients who die in the acute phase of their illness. The author cited a consensus publication which gave a MV duration of 21 days or more as an appropriate definition for “prolonged”. Cost savings from providing prolonged MV in settings other than acute hospitals was discussed. 1-year mortality was presented as the main measure of outcomes and was approximately 65% at the time of publication, indicating that prolonged MV has poor outcomes but is not entirely futile. Attempts to improve outcomes were discussed, these included earlier tracheostomy and improved post-hospital care. No interventions with proven benefits to outcome were identified.
Talmor *et al.* (2006) [28]	Systematic review	MV	This review was motivated by the observation that critical care is costly relative to much other care, imposing a significant burden on payers, and that demand is forecast to increase. The reviewers stated that an understanding of the cost-effectiveness of critical care is therefore required. The article also noted that no previous review of the cost-effectiveness of critical care had been conducted. The reviewers included nineteen studies which met their criteria. These criteria included the presentation of cost-per-life-year or cost-per-QALY CER. Many of the interventions considered, including MV, were found to be generally cost-effective. However, the reviewers noted that cost-effectiveness ratios are sensitive to the patient population studied and may change with time (as care improves). The article included some discussion of why there are so few cost-effectiveness studies in critical care. The authors suggested that this may be because only therapies with proven benefits may be subjected to CEA, and that the randomised controlled trials necessary to prove the benefits of critical care therapies are difficult to conduct and are consequently sparse.
Cooke (2012) [35]	Non-systematic review	MV	This was a non-systematic, but nonetheless fairly comprehensive, review of CEA studies of patients with acute respiratory failure. The article included tabulated CER data for different patient cohorts and treatment strategies. The focus of the paper was on how results from the literature should influence practice within ICU.
Wilcox *et al.* (2019) [29]	Systematic review	MV & ECMO	The review was motivated by the observation that, while the importance of CEA is widely acknowledged, it has been relatively little applied to critical care, a costly service which would appear well-suited to this kind of analysis. 97 articles were included in the review. Like Talmor *et al.*, the reviewers noted the relatively small numbers of relevant studies and suggested that the challenge of demonstrating therapies’ effectiveness may have been one contributing factor. The reviewers also noted that critical care patients typically receive many therapies, so isolating any one therapy for specific analysis is difficult. Most of the included studies which considered MV found it to be cost-effective. However, this was not the case for all patient cohorts. A single ECMO article was included [37], which found that ECMO is cost-effective. The reviewers noted that critical care is often supportive, and that a full understanding of its value would include considering its contribution as a facilitator of other procedures or surgeries.
Lazar (2020) [36]	Non-systematic review	ECMO	This article provided commentary on ECMO and the circumstances under which it may be life-saving and cost-effective. Lazar noted that ECMO mortality has decreased over time, being lower in 2013-2016 than it had been in 2008-2012. He noted that outcomes are poor for some patient groups including those suffering from dissection of the ascending aorta and irreversible cardiogenic shock, together with those aged >70 years or with high body mass index. Lazar also noted that post-intervention quality of life is an important factor in the determination of ECMO cost-effectiveness with one study reporting that >40% of ECMO survivors were unable to live independently or required ongoing health service input. He concluded by asserting the importance of prioritising ECMO therapy for the patients most likely to benefit from it.
Ontario Health (Quality) (2020) [30]	Systematic review	VA-ECMO	This review considered the effectiveness and cost-effectiveness of ECPR and of ECMO used to treat cardiogenic shock refractory to conventional management. Five studies were included for the cost-effectiveness assessment. None were directly relevant to the research question posed by the review. The researchers therefore built a Markov model using input parameters drawn from their clinical literature review. Their model used a five-year time horizon and concluded that for in-hospital and out-of-hospital cardiac arrest ECPR confers 1.08 and 0.69 life-years respectively. The incremental gains over conventional CPR were 0.56 and 0.24 life-years respectively.
Addison *et al.* (2022) [31]	Systematic review	ECPR	This review considered the cost-effectiveness of ECPR for out-of-hospital cardiac arrest. The reviewers identified four CEA studies which met their inclusion criteria [41,45,47,50]. All four studies found ECPR to be cost effective relative to comparators. The reviewers found the included studies to be of generally good quality but noted that they were heterogeneous in terms of the health systems within which they were based, this may affect the generalisability of the results.

**Table 2:** Included original research articles. A hyphen in the quality column indicates that the article did not present clinical value figures.

Reference, Country	Study Type	Technology	Quality	Patient Cohort	N	Main Outcome
Schmidt *et al.* (1983) [56] USA	Prospective	MV	Low	Patients who received ≥48 hours of MV.	137	The CER is high for older patients. But age alone is not sufficient to predict who will benefit from prolonged MV.
Thoner (1987) [58] Norway	Prospective	MV	Low	Patients requiring intermittent positive pressure ventilation for at least 48 hours.	249	Except for cancer patients, the CER for MV is favourable relative to other high-cost medical care.
Elpern *et al.* (1989) [72] USA	Prospective	MV	-	Patients aged ≥60 years who received MV for ≥3 days and did not spend time in surgical ICU.	95	Neither hospital length-of-stay nor duration of MV predict patient survival. Patients who survive three years tend to return to good levels of function.
Cohen *et al.* (1993) [57] USA	Prospective	MV	Low	Patients over the age of 80 who received at least three days of MV.	45	Cost-effectiveness was poor in this patient cohort.
Schapira (1993) [69] USA	Prospective	MV	-	Cancer patients admitted to ICU for non-postoperative care.	54	Most cancer patients admitted to ICU die in-hospital. Those discharged spend minimal time at home before dying.
Wachter *et al.* (1995) [55] USA	Prospective	MV	Low	Patients with severe respiratory failure, acquired immunodeficiency syndrome (AIDS) and *Pneumocystis carinii* pneumonia.	113	The CER compares unfavourably with those for other interventions.
Añón *et al.* (1999) [60] Spain	Prospective	MV	Moderate	Patients on long-term oxygen therapy (LTOT) with acute exacerbation of chronic obstructive pulmonary disease (COPD).	20	The outcomes and cost were poor. Several factors are significantly associated with mortality.
Mayer *et al.* (2000) [63] USA	Retrospective	MV	Moderate	Stroke patients requiring MV.	52	Treatment was cost-effective for extending life, but not cost-effective when quality of life was considered.
Dewar *et al.* (2000) [65] USA	Retrospective	MV	Low	Patients with a respiratory diagnosis requiring MV.	54,680	Treatment is cost-effective in most age ranges.
Hamel *et al.* (2000, 2001) [71,77] USA	Prospective	MV	-	Patients with acute respiratory failure who required MV support.	1005	Treatment was cost-effective for patients judged at admission to have a >50% probability of surviving at least 2 months. Treatment is cost-effective for patients with good short-term prognoses, but much less so for those without.
Douglas *et al.* (2002) [78] USA	Prospective	MV	-	Adult patients who received ≥24 hours of MV in hospital and who had not previously received MV at home.	538	Outcomes are poorer for patients who receive >96 hours of MV. A greater emphasis on post-discharge support is required, for patients’ families as well as patients themselves.
Cox, Carson, Govert *et al.* (2007) [75]	Model	MV	Low	Patients who received prolonged MV.	-	Cost-effectiveness varies substantially with age and with long- and short-term prognosis.
Cox Carson, Lindquist *et al.* (2007) [79] USA	Retrospective	MV	-	Adult patients who received ≥48 hours of MV.	817	Different definitions of prolonged MV capture significantly different patient populations.
Cooke *et al.* (2009) [80]	Model	MV	Low	Patients with acute lung injury.	-	Even a high-cost intervention to improve adherence to low-tidal volume ventilation protocols would be cost-effective.
Malmivaara *et al.* (2009) [70] Finland	Prospective	MV	Moderate	Neurosurgical patients who continue to require ventilatory support post-discharge.	346	Prolonged intensive care and step-down treatment of this patient group is clinically justified.
Peek *et al.* (2009,2010) [9,10] UK	Prospective	MV & ECMO (VV)	High	Patients with severe but potentially reversible respiratory failure.	180	ECMO is preferrable to conventional MV in patients with severe but potentially reversible respiratory failure.
Linko *et al.* (2010) [68] Finland	Prospective	MV	Moderate	Patients aged ≥16 years who received ≥6 hours of MV, non-invasive ventilation or continuous positive airway pressure support in ICU.	958	The CER is reasonable for this patient group, despite the relatively low health-related quality of life of survivors.
Hung *et al.* (2011, 2012) [61,62] Taiwan	Retrospective with prospective quality of life data collection	MV	Moderate	Patients who required at least 6 hours of MV for 21 days or more.	633	Prognosis is poor for this patient group. This could be used for communication to facilitate clinical decisions. Care for this patient group is also expensive. The cost should be considered at policy-level.
Park *et al.* (2014) [37] Brazil	Model	ECMO (VV)	Low	Severe ARDS patients in Brazil.	-	The cost-utility ratio for this patient group is potentially acceptable.
Lall *et al.* (2015) [54] UK	Prospective	MV	High	Patients aged ≥16 years predicted at admission to require at least 48 hours of MV. Patients with obstructive lung disease were excluded.	795	High frequency oscillatory ventilation (HFOV) has no economic advantage over conventional MV.
St-Onge *et al.* (2015) [41] Canada	Model	ECMO (VA)	Low	Patients in shock or cardiac arrest secondary to cardiotoxicant poisoning.	-	VA-ECMO may be cost effective for treating this patient group.
Chang *et al.* (2017) [42] Taiwan	Model	ECMO (VA)	Low	Patients receiving ECMO and a ventricular-assist device as a double bridge to heart transplantation.	-	Direct ventricular-assist device is more cost-effective than a double bridge with initial ECMO.
Kosiński *et al.* (2018) [43] Poland	Retrospective	ECMO (VA)	Low	Patients with severe hypothermia treated with VA-ECMO.	29	The cost of ECMO rewarming in this study was less than the reported cost of ECMO in other studies.
Saunders & Geogopoulos (2018) [73] UK & USA	Model	MV	Low	Patients receiving MV in an ICU.	-	Proportional assist ventilation is as cost effective as pressure support ventilation. The threshold for significant superiority was not reached.
Barrett *et al.* (2019) [38] Canada	Model	ECMO (VV)	Low	Adults with severe acute respiratory distress syndrome (ARDS).	-	VV-ECMO is cost-effective for this patient group.
Baston *et al.* (2019) [81]	Model	MV	-	Patients with acute respiratory distress syndrome (ARDS).	-	Interventions that increased use of proning in line with the assumptions made for this study would be cost-effective.
Bharmal *et al.* (2019) [45] USA	Retrospective	ECMO (ECPR)	-	Patients who received ECPR.	32	ECPR was cost-effective within the study context. Larger studies are required to assess whether cost-effectiveness is sensitive to other factors, such as patient characteristics.
Burišková *et al.* (2019) [46] Czech Republic	Retrospective	ECMO (ECPR)	Low	Patients who received ECPR at a single centre in Prague.	16	ECPR was cost-effective in this study, however further study is required for generalisable results. Cost-effectiveness would be improved if neurological outcome could be better predicted.
Dennis *et al.* (2019) [47]	Model	ECMO (ECPR)	Low	Patients in refractory cardiac arrest treated with ECMO.	-	ECMO support for this patient group is cost-effective.
Gravesteijn *et al.* (2019) [48]	Model	ECMO (ECPR)	Low	Patients treated with ECPR for in-hospital cardiac arrest.	-	ECPR is cost-effective relative to conventional willingness-to-pay thresholds
Kawashima *et al.* (2019) [49] Japan	Retrospective	ECMO (ECPR)	Moderate	Patients who received ECPR, were aged ≤75 years and arrived in hospital within 45 minutes of arrest	120	ECPR for patients with ventricular fibrillation or ventricular tachycardia was highly cost-effective, for patients with asystole or pulseless electrical activity cost-effectiveness was borderline
Bakker *et al.* (2020) [82]	Model	MV	Low	Patients receiving assistive modes of ventilation.	-	A technology to detect ineffective effort events could have the potential to lead to health and financial benefits.
Jäämaa-Holmberg *et al.* (2020) [44] Finland	Retrospective	ECMO (VA)	Moderate	CS patients eligible for heart transplantation.	102	The cost-utility of VA-ECMO use in a transplant setting is favourable.
Matsuoka *et al.* (2020) [50] Japan	Model	ECMO (ECPR)	Low	Out-of-hospital cardiac arrest patients, aged ≤75 years who arrived in hospital within 45 minutes of collapse or first call and received ECPR	-	ECPR is cost-effective for this patient group.
Ethgen *et al.* (2021) [39] France	Model	MV and ECCO_2_R	Low	Acute respiratory distress syndrome (ARDS) patients treated in an ICU.	-	Ultra-lung protective ventilation enabled by ECCO_2_R may provide a cost-effective survival benefit.
Doan *et al.* (2022) [51] Australia	Model	ECMO (ECPR)	Low	Adult patients with refractory out-of-hospital cardiac arrest.	-	The median ICER for ECPR is below commonly accepted willingness-to-pay thresholds.
Liao *et al.* (2022) [52] Taiwan	Retrospective	ECMO (all variants)	Low	Patients who received ECMO (all variants) at participating centres.	919	Ten variables were identified as predictors of in-hospital death. This should help identify patients with the best survival prospects.
Saunders *et al.* (2022) [74] Canada	Model	MV	-	Patients receiving MV in an ICU.	-	Proportional assist ventilation would be a cost-effective alternative to pressure support ventilation.
Agus *et al.* (2023) [40] UK	Prospective	ECCO_2_R	Moderate	Patients aged ≥16 years with an acute and potentially reversible cause of moderate to severe respiratory failure.	412	ECCO_2_R is not a cost-effective alternative to conventional management.
Oude Lansink-Hartring *et al.* (2023) [53] The Netherlands	Prospective	ECMO (all variants)	Moderate	All patients receiving ECMO (all variants) at the participating centre.	428	ECMO may be cost-effective, but conclusions are weakened by the number of patients lost to follow-up.

All the included review articles considered therapies’ cost-effectiveness, not specifically their clinical value. Systematic reviews published in 2006 [28] and 2019 [29] considered the cost-effectiveness of ICU care in general. The latter considered both MV and ECMO, concluding that both therapies are generally cost-effective. The former did not consider ECMO, having been conducted before its use became widespread. Both reviews observed that ICU cost-effectiveness research is sparse when compared to other specialties, noting that demonstrating ICU therapies’ cost effectiveness is often challenging, and that consequently few ICU therapies are supported by this kind of evidence, which is often considered a pre-requisite for cost-effectiveness research.

The two other included systematic reviews considered the cost-effectiveness of VA-ECMO (including ECPR) [30,31]. Both noted that the literature in this area is heterogeneous and that conclusions may not be generalisable.

Five non-systematic reviews were included. Four considered whether MV is cost-effective [[32], [33], [34], [35]], all focussing on the USA healthcare context. These articles also considered factors which may affect MV cost-effectiveness, these included the age and disease profiles of the patient population [35] and the staffing model of the ICU [33]. One non-systematic review considered the clinical- and cost-effectiveness of ECMO [36]. The author discussed the dependence that exists between ECMO cost-effectiveness and the population upon whom it is used, concluding that ECMO should be prioritised for those patients most likely to benefit from it. Although these discussions were concerned with cost-effectiveness rather than clinical value, the identified dependencies may also apply to clinical value, the denominator of the CER equation.

The included MV original research articles spanned a longer time-period than those for ECMO, with the earliest for MV appearing in 1983, and the earliest for ECMO in 2009. Up until 2012 most MV studies (14 of 18) were concerned with the overall cost-effectiveness of MV therapy. Only six articles have been published since 2012, these all compared novel MV techniques or technologies with conventional MV, rather than investigating overall cost-effectiveness. By contrast, most relevant ECMO studies (13 of 18) included a comparator therapy, and publication rate increased over time with three relevant studies published between 2009 and 2016, and fifteen between 2017 and 2023.

Of the ECMO studies, three considered VV-ECMO [10,37,38], two considered ECCO_2_R [39,40], four considered VA-ECMO [[41], [42], [43], [44]], seven considered ECPR [[45], [46], [47], [48], [49], [50], [51]] and the remaining two considered all ECMO patients seen at participating centres [52,53].

Of the 24 MV studies, 12 were prospective (including two RCTs [9,54]), 5 (21%) were retrospective and 7 (29%) were models. For ECMO, modelling studies were more common, comprising 9 of the 18 included studies (50%), 6 (33%) were retrospective and only 3 (17%) were prospective (of which two were RCTs [9,40]). Two studies were included in both the MV and ECMO analyses [9,39].

### 3.2 Study quality

The quality assessments are presented in

Table 2. These assessments consider the quality of these studies with respect to the present article's objectives – assessment of MV and ECMO value such as can be used to inform local medical equipment management decisions. None of the included studies shared these objectives. The quality assessments presented here do not reflect the quality of included articles with respect to their own objectives, or an assessment of the strength of their own conclusions.

Two articles, both reporting the results of RCTs [9,54], were classified as “high” quality.

Of the prospective studies, four presented non-quality-assessed life expectancy data [[55], [56], [57], [58]] and were classified as “low” quality. All others were classified as “moderate” quality. Prospective study designs tended to enable the collection of high-quality quality of life data. Of the prospective studies which attempted quality of life assessment, only one used a method other than the EuroQoL EQ-5D instrument [59] (an accepted gold standard for quality-of-life assessment in critical care [27]) [60]. This study was published before the EQ-5D's acceptance as a gold standard methodology.

The most consistent difference between prospective and retrospective studies was that the latter tended to use lower-quality quality of life assessment methodologies. This may be because quality of life data is not typically gathered for routine clinical care, whose records form the basis of most retrospective data sets. EQ-5D data was however routinely collected at the centre used by Jäämaa-Holmberg *et al.* [44]. Hung *et al.* [61,62] also had access to EQ-5D data, having prospectively collected it from a subset of the patients whose data was included in their retrospective data set.

Retrospective researchers used a variety of strategies to address the lack of quality-of-life data. Mayer *et al.* [63] assigned quality of life utility values derived from the Rankin scale (used to score the functional independence of stroke survivors), based on previously published data [64]. Dewar *et al.* [65] assigned a utility of 1 for every year of life before the age of 80, and a score of 0.81 for every year thereafter. This strategy was derived from a previous study which found that, on average, hospital inpatients aged ≥80 years assigned a utility of 0.81 to their current health state [66]. Of note, this study was not specific to the patient cohort considered by Dewar *et al.* and excluded some patient groups relevant to the latter study (e.g., patients aged <80 years). Except Jäämaa-Holmberg *et al.,* all retrospective ECMO studies assigned quality of life utility values using the Cerebral Performance Categories (CPC) scale, used to score neurological function [46,49,52]. Burišková *et al.* [46] and Liao *et al.* [52] did this using a technique published by Stiell *et al.* [67] while Kawashima *et al.* [49] inferred utility values using other published data. These alternative strategies tended to derive quality of life scores using functional data only, this is different from gold standard tools which patient preferences factors also.

All modelling studies were classified as “low” quality. These studies usually drew model input parameters from multiple other studies, each considering a different patient cohort with different characteristics. This makes it difficult to understand how the model outputs would apply to a real patient cohort.

A common limitation of both prospective and retrospective studies related to life expectancy calculations. For a small minority of studies, the deaths of all participants occurred during the follow-up period (e.g., Wachter *et al.* [55]). For all other studies, it was necessary to model the life expectancy of surviving participants. Many studies assumed that the life expectancy of participants who survived the follow-up period had the same life expectancy as age- and sex- matched members of the general population [9,43,44,49,56,57,65,68]. However, other studies noted that ICU survivors tend to have impaired life expectancy and quality of life relative to controls [35,62,63,68,69]. Studies which assume life expectancy equivalent to the general population are likely to overestimate life expectancy and therefore also overestimate the clinical value of MV. Of note, this limitation applied to the CESAR RCT [9,10], which was nonetheless classified as “high” quality due to strength in other areas.

Overall, the quality of MV studies was broadly similar to that for ECMO studies, although with a slightly greater proportion of moderate and high-quality studies for MV, likely in part because a greater proportion of the MV studies were prospective in design.

### 3.3 Clinical value and clinical value per unit time

**Fig. 2:**
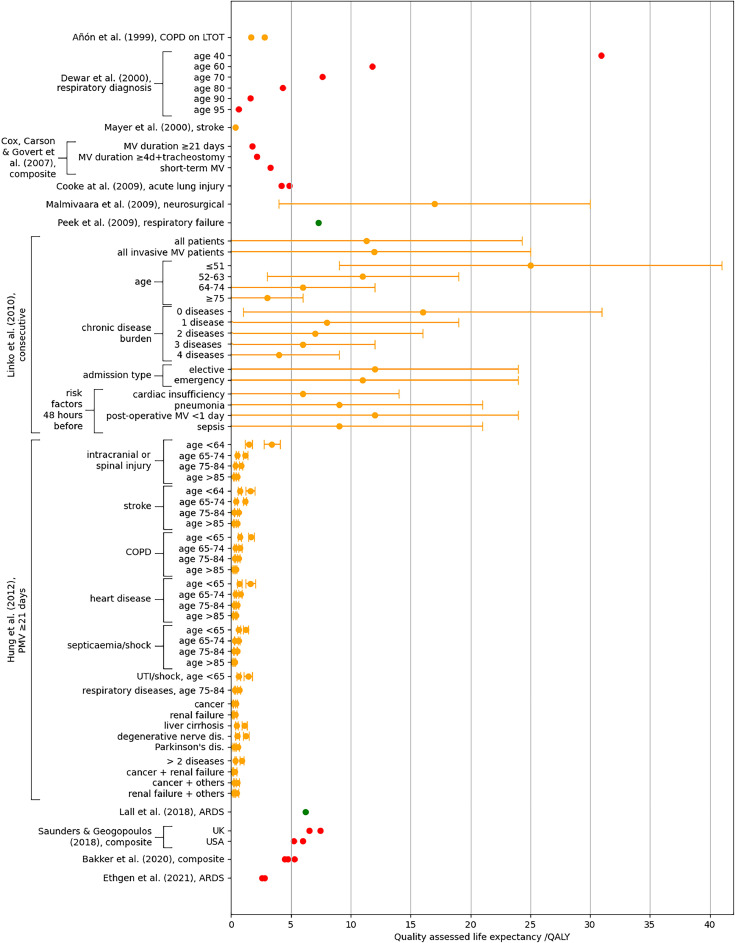
MV QALE values from included studies. The label on the vertical axis shows the author's name, year of publication and a short descriptor of the patient cohort; “consecutive” indicates that the cohort consists of consecutive patients seen at one or more centres, “composite” indicates a modelled cohort not intended to represent a specifically defined patient cohort. The marker colour denotes the quality of the analysis, green denotes high quality, orange denotes moderate quality, and red denotes low quality. Error bars show the standard deviation, where available. Where more than one marker appears for a single category, this indicates that the article published multiple values (e.g., Hung et al. published separate values for patients cognitively able to complete the quality-of-life questionnaire and for those unable to do so), consult the supplemental material for further details. Data are presented by order of publication, with the earliest at the top of the plot. Acronyms: ARDS = acute respiratory distress syndrome, COPD = chronic obstructive pulmonary disease, LTOT = long-term oxygen therapy, PMV = prolonged mechanical ventilation, UTI = urinary tract infection.

**Fig. 3:**
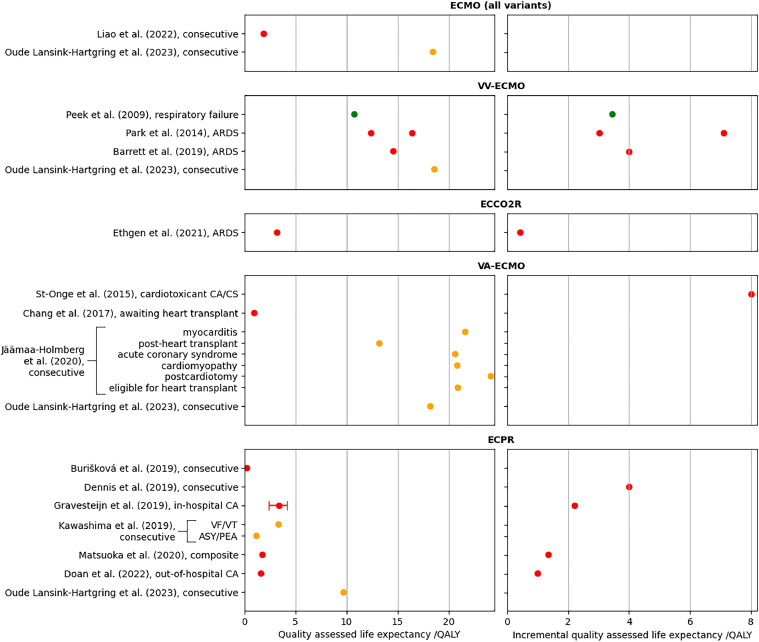
ECMO QALE values from included studies. The label on the vertical axis shows the author's name, year of publication and a short descriptor of the patient cohort; “consecutive” indicates that the cohort consists of consecutive patients seen at one or more centres, “composite” indicates a modelled cohort not intended to represent a specifically defined patient cohort. The plots on the left show the total QALE, the plots on the right show the incremental QALE with respect to the comparator technology, where available. The marker colour denotes the quality of the analysis, green denotes high quality, orange denotes moderate quality, and red denotes low quality. The error bars for the Gravesteijn et al. data show the credibility interval for the QALE estimate. Data are grouped by the different ECMO variants, the “ECMO (all variants)” data is from articles which presented results which included data from more than one ECMO variant. Within each group, data are presented by order of publication. The Agus et al. [40] data is omitted, as this group used a one-year horizon for their calculations, so their results are not comparable to those of other studies, which used lifetime horizons. Acronyms: ARDS = acute respiratory distress syndrome, ASY = asynchrony, CA = cardiac arrest, CS = cardiogenic shock, PEA = pulseless electrical activity, VF = ventricular fibrillation, VT = ventricular tachycardia.

All QALE values for MV are shown in Fig. 2. QALE and incremental QALE values for ECMO are shown in Fig. 3. The numeric values, together with values for QALE per unit time and incremental QALE per unit time, are also available in the supplemental material.

Only Hung *et al.* [61,62] explicitly sought to publish QALE values. Other articles sought to publish CER or ICER values, but also published the QALE values which were used in the calculations of these parameters, but whose evaluation was not the primary purpose of the study.

Different MV studies considered different patient groups, so results are not all comparable. Hung *et al.*, [61,62] who considered only patients who received MV for at least 21 days, reported the lowest QALE values. These ranged from 0.1 QALY (for patients aged >85 years, with septicaemia or shock) to 3.4 QALY (for patients aged <64 years, with an intracranial or spinal injury). The model described by Dewar *et al.* [65] produced the highest QALE value, of 57 QALY for 20-year-old patients with a respiratory diagnosis. The two studies assessed as being of highest quality gave mean QALE values of 7.3 QALY (for patients with severe but potentially reversible respiratory failure) [10], and 6.2 QALY (for acute respiratory distress patients) [54]. A minority of articles reported the MV duration data necessary to estimate the value conferred per day of treatment (these data are given in the supplemental material where available). The values ranged between 0.05 QALY/day (for the stroke patient cohort considered by Mayer *et al.*) [63], to approximately 2.85 QALY/day (for the neurosurgical cohort considered by Malmivaara *et al.*) [70].

The reported QALE values for VV-ECMO (used as an escalation therapy for patients refractory to conventional MV) were generally slightly higher than equivalent MV values, ranging from 10.8 [10] to 18.6 QALY [53]. For ECCO_2_R (used as an adjunct to MV) Ethgen *et al.* [39] reported a QALE figure of 3.2 QALY, and an incremental QALE of 0.4 QALY.

The patient cohorts, and destination therapies, considered by VA-ECMO articles were diverse, making comparison difficult. This ECMO variant delivered some of the highest reported QALE values, with Jäämaa-Holmberg *et al.* [44] reporting values greater than 20 QALY for all diagnostic groupings except patients who developed cardiogenic shock having already received a heart transplant. Oude Lansink-Hartgring *et al.* [53] reported a similarly high value of 18.2 QALY. Chang *et al.* [42] reported a much lower figure of 1.0 QALY for patients receiving bridging therapy while awaiting a heart transplant.

The QALE values reported by ECPR articles were all less than 3.5 QALY, except for that reported by Oude Lansink-Hartgring *et al.* [53] (9.7 QALY).

The incremental QALE values reported by ECPR studies (which all used conventional CPR as the comparator) were generally similar to the absolute QALE figures, indicating that QALE for conventional CPR patients is usually low. Three VV-ECMO studies reported incremental QALE values (each with MV as the comparator), the reported values were 3.4 QALY [10], 4.0 QALY [38], and 3.0 QALY for patients given a 40% chance of survival at admission and 7.1 for patients given a 60% chance of survival at admission [37].

All prospective and retrospective ECMO articles, except one [46], reported ECMO duration, while only two modelling studies reported these data [39,47]. Overall, duration data were more often available for ECMO than for MV. The absolute QALE/day figures (available in the supplemental material) were generally higher for ECMO than for MV, ranging from 0.12 QALY/day [10] to 10.3 QALY/day [44]. Incremental QALE/day values were available for only for the CESAR trial of VV-ECMO (0.04 QALY/day) [10], the Dennis *et al.* model for ECPR (1.05 QALY/day) [47], and the Ethgen *et al.* model for ECCO_2_R (0.03 QALY/day) [39]. These incremental values are lower than the typical QALE/day figures reported for MV.

## 4 Dependencies

**Fig. 4:**
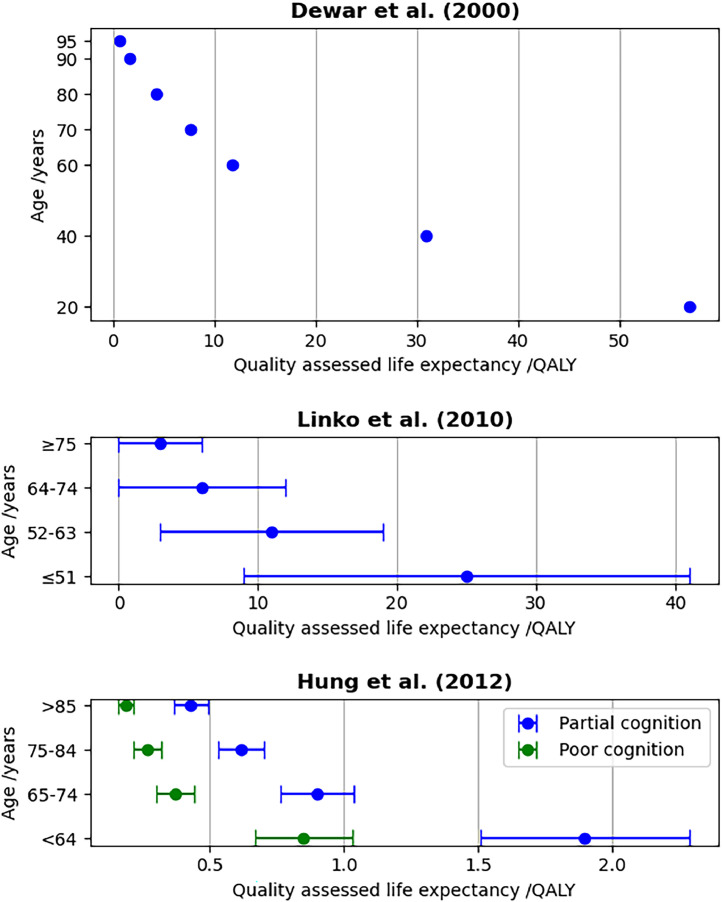
Plots of the dependence of QALE on age, extracted from tabulated data presented by Dewar et al. [65], Linko et al. [68] and Hung et al. [61]. The Hung et al. data is aggregated data for patients with chronic obstructive pulmonary disease (COPD), heart disease, intracranial or spinal injury, septicaemia or shock, or stroke. “Partial cognition” refers to patients cognitively able to complete a quality-of-life questionnaire, “poor cognition” refers to those unable to do so. Error bars show standard deviations, where available.

Several MV articles presented analyses for different age groups [58,61,65,68], those which presented QALE values are shown in Fig. 4. QALE was greater for younger patients in all cases. No authors tested the statistical significance of this difference. The relationship was non-linear, with the effect diminishing with increasing age. Several authors commented that age is not sufficient to predict clinical outcome [56,57,71]. No age group analysis was presented by any of the ECMO articles.

Hung *et al.* [61] presented separate MV QALE analyses for different diagnostic groupings (plotted in Fig. 2). Schmidt *et al.* [56] and Thoner [58] also published analyses of (non-quality assessed) life expectancy broken down by diagnosis. None of these authors analysed the statistical significance of the differences between groupings, however their results appear to show a consistent difference, and other authors also assumed a relationship between diagnosis and MV cost-effectiveness [28,57,72].

No analysis by diagnostic grouping was presented for VV-ECMO or EECO_2_R. Jäämaa-Holmberg *et al.* [44] did present VA-ECMO QALE data partitioned by cause of cardiogenic shock or cardiac arrest. In their study of ECPR cost-effectiveness Kawashima *et al.* [49] also partitioned by presenting heart rhythm. Neither group analysed the statistical significance of the difference between groupings. In his review of the ECMO literature Lazar [36] also expressed the view that ECMO, while cost-effective for some patients, is unlikely to be cost-effective for others, such as those with aortic dissection or irreversible cardiogenic shock, and older patients.

Several MV articles considered QALE dependencies on factors other than age and diagnosis. Linko *et al.*, considered the number of chronic diagnoses, finding that QALE diminished with increasing numbers of chronic diagnoses, with a particularly large decrease in QALE for the first chronic diagnosis (Fig. 2). Dewar *et al.* [65] used a data set covering the five-year period from 1992-1996 (inclusive) to produce polynomial (cubic) models of the relationship between hospital survival and patient age, producing a different model for each calendar year within their study. Their models suggested a year-on-year improvement in hospital survival. The authors noted that this improvement may be due to in part to improving care, and in part due to increased use of “do not resuscitate” orders resulting in fewer patients with poor prognosis being placed on MV. In their systematic review, Talmor *et al.* [28] also discussed the time-dependent nature of cost-effectiveness ratios, noting that both the cost and effectiveness of a therapy is likely to change with time. Shorr [33] noted that “closed” ICU staffing models, using formal treatment protocols and staffed by full-time intensivists, may improve treatment outcomes and simultaneously reduce costs. Shorr also noted that the cost-effectiveness of these factors is not easily studied. Indeed, no empirical studies into these factors were identified in this review. Cooke [35] also noted that different staffing arrangements and use of long-term acute care facilities can impact costs and, to a lesser extent, patient outcomes. Finally, it was implicit in several articles that the QALE conferred by a technology may vary between nations. These included Saunders & Geogopoulos [73] who presented separate analyses for the UK and USA (and, in a further publication, for Canada [74]), and other articles which explicitly sought to present nation-specific analyses [30,37].

## 5 Discussion

This review was motivated by a desire to investigate whether it is possible for a healthcare provider to use published data to derive estimates of the clinical value likely to be conferred by MV and ECMO equipment in their context.

A limited number of the reviewed articles presented clinical value estimates. Except for the articles by Hung *et al.* [61,62] (which specifically sought to present QALE values) these articles aimed to present CER or ICER values, and the evaluation of clinical value estimates was incidental to this goal. Other articles which presented CER or ICER values did not present the QALE values used in their calculations. This reduced the amount of data available for the present study and the strength of the conclusions that could be drawn.

Articles which did present clinical value figures differed profoundly in terms of the methods used and the patient populations considered. As such, they were generally neither equivalent, nor directly comparable. The most significant methodological distinction was between empirical studies (which drew inferences from a single data set) and modelling studies (which relied on input parameters drawn from multiple sources). A limitation of the empirical approach was that the conclusions applied only to patient populations and operational circumstances similar to those considered by the study. A limitation of the modelling approach was that input parameters were typically drawn from a range of different studies, conducted with different patient populations and in different operational contexts making it difficult to determine which real patient populations and contexts they apply to.

Modelling studies have become more common with time and constituted a correspondingly high proportion of the included ECMO studies, most of which were published in the last five years. The literature search also returned fewer prospective studies for ECMO than for MV. Prospective studies tended to produce the highest quality data and were also more likely to present subgroup analyses and other parameters of interest than were modelling studies. There was therefore much less clinical value data available for ECMO than for MV, and the data which did exist tended to be of poorer quality.

This relative sparsity of ECMO data is exacerbated by the fact that those publications which do exist are split between the VV-ECMO, VA-ECMO, ECCO_2_R and ECPR variants. These therapies involve the same equipment but are applied to different patient populations. Analyses of the value conferred should consider them separately. Overall, this will mean that local estimates of the clinical value conferred by these technologies, made using published data, are likely to be less accurate for ECMO than for MV.

When assessing the value offered by medical equipment it is important to consider the alternatives to that equipment. Most MV articles, implicitly or explicitly, assumed that no alternative therapy exists. Patients eligible for MV, but from whom MV was withheld, were therefore considered to have negligible life expectancy and negligible cost associated with their care, an assumption known as the “zero-cost zero-life” assumption. An exception was Cox, Carson, Govert *et al.* [75], who modelled the QALE associated with MV withdrawal, assigning it a value of 0.029 QALY (equivalent to 11 days’ life in perfect health). This low value suggests that the zero-cost zero-life assumption is a broadly appropriate approximation for MV. Under this assumption, the value conferred by MV equipment is equivalent to the value of the total life lived by survivors after the therapy. QALE estimates were low (<1 year) for some patient groups (e.g., those receiving prolonged MV or suffering from stroke), but greater QALE estimates were otherwise typical (Fig. 2).

In contrast to MV, alternatives usually exist to ECMO therapy, the zero-cost zero-life assumption is therefore less often appropriate. Conventional MV is an alternative to VV-ECMO and ECCO_2_R augmented MV, while conventional CPR is an alternative to ECPR. In these cases, if ECMO equipment were unavailable then only the incremental value delivered using ECMO equipment would be lost (provided that the alternative therapies were available). Most articles considering these made direct comparisons with conventional alternatives and presented incremental results (Fig. 3). VA-ECMO articles were less likely to present comparative results, suggesting that the zero-cost zero-life assumption may sometimes be appropriate for this therapy.

Another objective of this work was to estimate the value conferred by these technologies per unit duration of use, this required duration-of-use data. These data were more often available for ECMO than for MV but were seldom available in combination with incremental QALE values (which, for this technology, are generally more relevant than absolute values, as discussed above). Calculation of the incremental QALE conferred per unit treatment duration is therefore generally not possible. For both technologies duration-of-use may be sensitive to factors which vary between healthcare providers, such as local procedures and pressure on ICU bedspace capacity. For a given provider, more accurate estimates may be derivable from local records than from published data.

A final objective of this work was to identify dependencies which are predictive of MV and ECMO conferred clinical value. Published MV data, particularly from empirical studies which included subgroup analyses, did reveal some dependencies. QALE is likely to depend on diagnosis and, according to one group, on the total number of chronic diagnoses [68]. QALE also appeared to be dependent on patient age. The details of this dependency should be interpreted with caution; for patients who survived studies’ follow-up periods QALE was nearly always estimated using a model which took patient age as an input. The age/QALE relationships presented by analyses are therefore partly determined by the details of these models rather than solely by the gathered empirical data.

Few of the reviewed ECMO articles included subgroup analyses, so elucidation of dependencies is difficult. For ECPR performed on cardiac arrest patients, it does appear that mean QALE is greater for those presenting with ventricular fibrillation or ventricular tachycardia than those presenting with non-shockable rhythms, a conclusion presented by Kawashima *et al.* [49] and supported by evidence of poorer ECPR clinical effectiveness for the latter group [24,76]. In his review, Lazar pointed out that ECMO clinical effectiveness is influenced both by age and diagnosis [36]. Any factor affecting clinical effectiveness would be expected to have a corresponding effect on cost-effectiveness and clinical value conferred, however the reviewed articles did not quantify the size of this effect.

Several authors discussed other dependencies in narrative sections of their articles, particularly for MV. These included the ICU staffing model (i.e., whether the ICU uses a “closed” or “open” model), the country in which treatment occurs, and time – with treatment outcomes generally expected to improve with time. The sizes of these effects were not quantified.

In conclusion, estimates of the clinical value conferred by both MV and ECMO are available in the literature. The magnitude of this clinical value has dependencies, particularly on characteristics of the patient population upon whom the therapy is used. The same equipment would therefore be expected to confer different clinical value for healthcare providers serving different patient populations. Data quantifying the importance of some dependencies is available for MV but is largely unavailable for ECMO. Local estimates of the value conferred by ECMO equipment are therefore likely to carry greater uncertainty than equivalents for MV.

These local estimates of the value conferred by MV and ECMO equipment could be used to predict the impact of decisions which affect availability of this equipment. These could include decisions to procure new equipment, to decommission existing equipment, or decisions which affect equipment downtime (e.g., by affecting the resources available for maintenance). These local value estimates could also be used to inform decisions which involve prioritising MV and ECMO relative to one another. Use of data to inform decisions in this way would help healthcare providers maximise the benefit derived from the finite equipment management budgets available to them. Further research to quantify the clinical value conferred by other equipment categories would broaden the pool of equipment whose clinical value could be systematically estimated and compared and would permit data-informed prioritisation decisions for a greater range of equipment types.

## CRediT authorship contribution statement

**David Stell:** Conceptualization, Data curation, Formal analysis, Funding acquisition, Investigation, Methodology, Project administration, Software, Visualization, Writing – original draft. **Dr Man Ting Kwong:** Writing – review & editing. **Robert Megwa:** Writing – review & editing. **Dr Tom Bashford:** Supervision, Writing – review & editing. **Dr. Emmanuel Akinluyi:** Conceptualization, Funding acquisition, Supervision, Writing – review & editing. **Prof. P. John Clarkson:** Supervision, Writing – review & editing.

## Declaration of competing interest

None declared.

## Appendix Supplementary materials

Image, application 1

Image, application 2

Image, application 3
