# Supplementary material for: A review of the clinical value of mechanical ventilators and extracorporeal membrane oxygenation (ECMO) equipment
Source: IPEM Transl. 2024 Dec;12:None. doi: 10.1016/j.ipemt.2024.100031 (PMC11647597; doi:10.1016/j.ipemt.2024.100031)
Supplement: Supplementary file 1 [file mmc1.docx]

Supplemental Material

A Review of the Clinical Value of Mechanical Ventilators and Extra Corporeal Membrane Oxygenation (ECMO) Equipment

# Method

## Literature Search

The search terms used for the literature search are given below:

| Mechanical Ventilation | ECMO |
| --- | --- |
| (  (mechanical ventilat*)  ) AND (  (QALY*) OR  (Quality Assessed Life Year*) OR  (Quality-Assessed Life Year*) OR  (Quality-Assessed Life-Year*) OR  (Quality Assessed Life-Year*) OR  (Quality Adjusted Life Year*) OR  (Quality-Adjusted Life Year*) OR  (Quality-Adjusted Life-Year*) OR  (Quality Adjusted Life-Year*) OR  (cost-effective*) OR  (cost effective*) OR  (cost-benefit) OR  (cost benefit) OR  (cost-utility) OR  (cost utility) OR  (Health Technology Assessment) OR  (HTA)  ) | (  ("extracorporeal membrane oxygenation") OR  ("extra corporeal membrane oxygenation") OR  ("extra-corporeal membrane oxygenation") OR  (ECMO) OR  ("extracorporeal life support") OR  ("extra corporeal life support") OR  ("extra-corporeal life support") OR  (ECLS) OR  ("extracorporeal carbon dioxide removal") OR  ("extra corporeal carbon dioxide removal") OR  ("extra-corporeal carbon dioxide removal") OR  (ECCO2R) OR  ("extracorporeal pulmonary resuscitation") OR  ("extra corporeal pulmonary resuscitation") OR  ("extra-corporeal pulmonary resuscitation") OR  (ECPR)  ) AND (  (QALY*) OR  ("Quality Assessed Life Year*") OR  ("Quality-Assessed Life Year*") OR  ("Quality-Assessed Life-Year*") OR  ("Quality Assessed Life-Year*") OR  ("Quality Adjusted Life Year*") OR  ("Quality-Adjusted Life Year*") OR  ("Quality-Adjusted Life-Year*") OR  ("Quality Adjusted Life-Year*") OR  ("cost-effective*") OR  ("cost effective*") OR  ("cost-benefit") OR  ("cost benefit") OR  ("cost-utility") OR  ("cost utility") OR  ("Health Technology Assessment") OR  (HTA)  ) |

## Clinical Value per Unit Time

The algorithm used to estimate the mean therapy duration, where the median and quartiles were provided, was implemented using the Python programming language, version 3.10.9, including the SciPy library, version 1.10.0. The algorithm calculates the skew normal, gamma and Weibull distributions defined by the median and quartiles, plots them, and calculates their respective means. The user can use the plots to identify the distribution which best matches the data. The algorithm is available from the address below:

<https://github.com/David-S2/distributions-from-quartiles>.

# Results

## QALE Gain per Unit Time


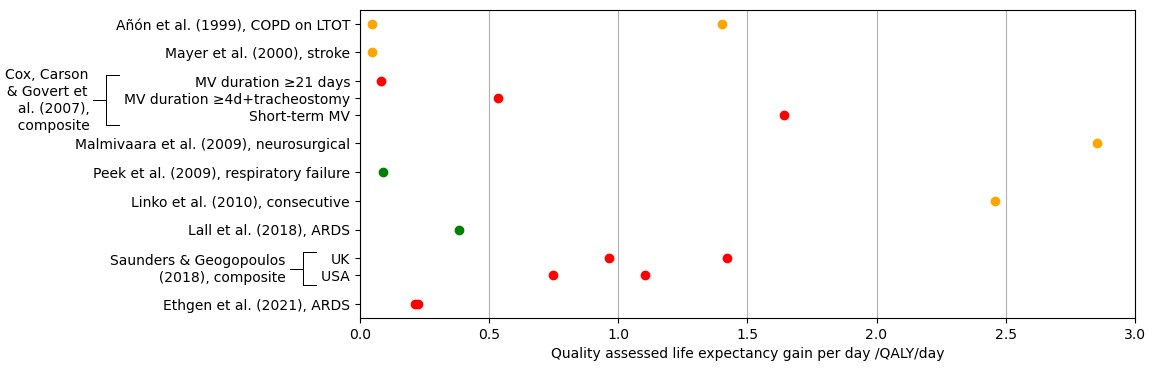


Figure 1 – MV QALE conferred per unit time from included studies. The label on the vertical axis shows the author’s name, year of publication and a short descriptor of the patient cohort; “consecutive” indicates that the cohort consists of consecutive patients seen at one or more centres, “composite” indicates a modelled cohort not intended to represent a specifically defined patient cohort. The marker colour denotes the quality of the analysis, green denotes high quality, orange denotes moderate quality, and red denotes low quality. Where more than one marker appears for a single category, this indicates that the article published multiple values, consult the complete table of QALE values for further details. Data are presented by order of publication, with the earliest at the top of the plot.


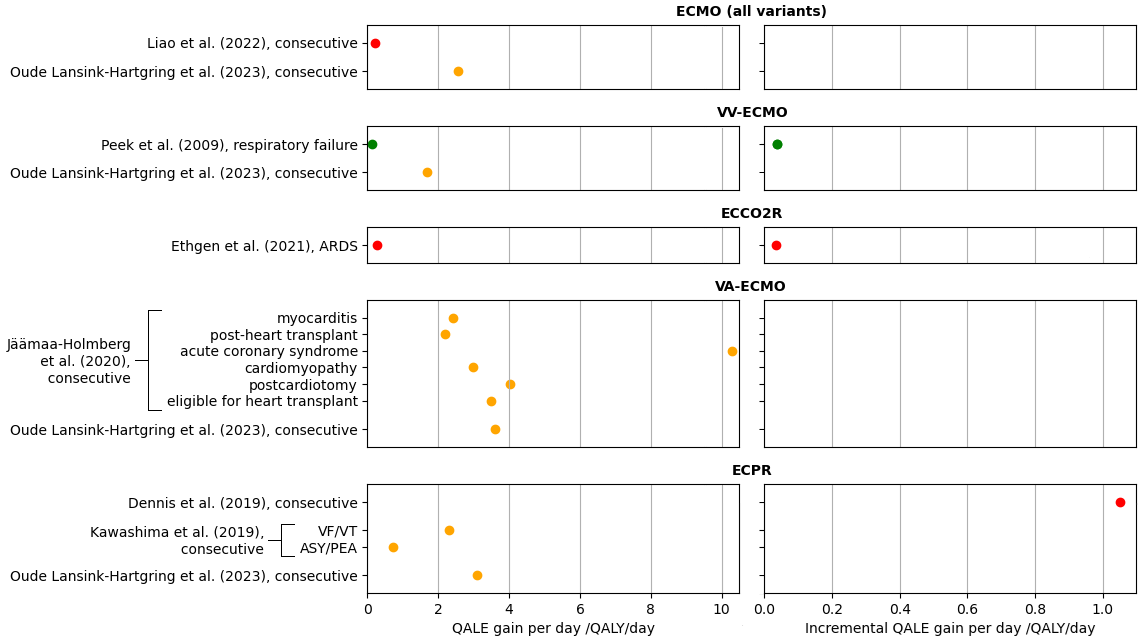


Figure 2 - ECMO QALE per unit time from included studies. The label on the vertical axis shows the author’s name, year of publication and a short descriptor of the patient cohort; “consecutive” indicates that the cohort consists of consecutive patients seen at one or more centres. The marker colour denotes the quality of the analysis, green denotes high quality, orange denotes moderate quality, and red denotes low quality. Data are grouped by the different ECMO variants, the “ECMO (all variants)” data is from articles which presented results which included data from more than one ECMO variant. Within each group, data are presented by order of publication. The Agus et al. data is omitted, as this group used a one-year horizon for their calculations, so their results are not comparable to those of other studies, which used lifetime horizons.
